# A validation study of the Intentional Nonadherence Scale among people with type 2 diabetes in the United Kingdom

**DOI:** 10.1111/dme.70040

**Published:** 2025-04-05

**Authors:** Vivien Teo, Anna Hodgkinson, John Weinman, Mark Chamley, Kai Zhen Yap

**Affiliations:** ^1^ Institute of Pharmaceutical Sciences, King's College London London UK; ^2^ Department of Pharmacy National University of Singapore Singapore Singapore; ^3^ Lambeth Diabetes Intermediate Care Team Guy's and St Thomas' NHS Foundation Trust London UK

**Keywords:** diabetes mellitus, medication adherence, psychometrics, type 2

## Abstract

**Aim:**

To examine the psychometric properties of the Intentional Nonadherence Scale (INAS) among people with type 2 diabetes mellitus (PwT2D) in the United Kingdom.

**Methods:**

This validation study recruited 260 PwT2D at diabetes intermediate care team clinics in London. Thirty of them participated in the test–retest reliability analysis in 2–4 weeks, while 124 were followed up in 3–6 months for the predictive validity analysis. The psychometric evaluation also comprised internal reliability, structural validity and construct validity that assessed the relationship between the INAS and other established measures, such as the Medication Adherence Report Scale‐5 (MARS‐5), Beliefs about Medicine Questionnaire (BMQ)‐specific, Brief Illness Perception Questionnaire (BIPQ), Patient Health Questionnaire‐2 (PHQ‐2) and glycated haemoglobin (HbA1c).

**Results:**

Exploratory factor analysis revealed four factors, namely ‘Resisting illness’, ‘Resisting medication’, ‘Testing treatment’ and ‘Sensitivity to medication’. All INAS factors demonstrated high internal reliability (Cronbach's alpha = 0.92–0.96). Their test–retest reliability varied between <0.001 and 0.92. Construct validity was demonstrated by its relationship with other measures, including its negative correlations with medication adherence and positive correlations with medication concerns. Significant correlations were also found with HbA1c, as well as with PwT2D's perceptions of diabetes consequences, treatment control, identity and emotional responses to diabetes. ‘Testing Treatment’ showed a trend towards statistical significance with adherence in 3–6 months (coefficient = −0.34, *p* = 0.09).

**Conclusions:**

The INAS performed well on a number of psychometric properties in this study. It may be a helpful tool for clinicians in identifying specific drivers of intentional nonadherence among PwT2D.

**What's new?**

- Medication nonadherence is prevalent in people with type 2 diabetes and limits the effectiveness of medications.
- This study evaluated the psychometric properties of the Intentional Nonadherence Scale (INAS), designed to explore reasons why people may choose not to take their medications.
- The INAS performed well on a number of psychometric properties, including its associations with glycated haemoglobin (HbA1c) and other self‐reported measures.
- The INAS may be a helpful tool for clinicians in identifying specific drivers of intentional nonadherence among people with type 2 diabetes.

## 1 INTRODUCTION

There had been several significant advancements in the development of diabetes medications in the United Kingdom (UK). New antidiabetic medications have emerged, including sodium glucose co‐transport 2 inhibitors, which also reduce cardiovascular and renal risks,^1^ and glucagon‐like peptide‐1 receptor agonists, which have some cardiovascular benefits.^2^ Medications are key in diabetes management. However, their effectiveness may be limited by current adherence levels.

Nonadherence to diabetes medications was estimated at about 50%.^3^ The World Health Organisation proposed that improving the effectiveness of medication adherence intervention may have a more significant impact on health than advancing specific medical treatments.^4^ Glycated haemoglobin (HbA1c) decreased by 2 mmol/mol (0.2%) for every 10% increase in medication adherence (*p* < 0.0001).^5^ All‐cause hospitalisation (Odds ratio, OR 0.83, *p* < 0.01) and mortality (OR 0.75, *p* < 0.01) significantly decreased with a 25% improvement in medication adherence.^6^

Adherence measures, such as proportion of days covered, are proxies for adherence levels; however, they do not elicit reasons for unintentional and intentional nonadherence in people with type 2 diabetes (PwT2D). Unintentional nonadherence happens when people lack the resources and capability to adhere to their medications, while intentional nonadherence happens when people choose not to take their medications as prescribed. Recent research proposed that unintentional nonadherence, such as forgetfulness, may be influenced by factors associated with intentional nonadherence, such as medication beliefs.^7^ Unintentional and intentional nonadherence may overlap, indicating that interventions should address the underlying causes of intentional nonadherence, instead of focusing solely on unintentional nonadherence, which can involve complex factors such as beliefs and motivations.^7^ This may also explain why unidimensional interventions, such as reminders which focus on unintentional nonadherence, have been ineffective for improving adherence.^8^

The Intentional Nonadherence Scale (INAS) was designed to investigate people's reasons for deciding not to take medications as prescribed,^9^ including the impact medication has on people's self‐perception and people's attempts to get away with less or no medication.^9^ Its development was informed by qualitative literature and psychological concepts.^9^ The INAS demonstrated promising psychometric properties in earlier validation studies among people with hypertension,^9^ cancer,^9^ gout,^9^, ^10^ and chronic pain.^11^ Additionally, it has been translated into Mandarin and adapted into local English for testing among PwT2D in Singapore, a developed, multiracial, multilinguistic Asian country, where it performed well on key psychometric indicators.^12^ While the Singapore INAS study findings may be relevant to PwT2D in the UK due to the same medical condition under study, more investigation is required. Differences in cultural contexts, languages, healthcare systems and population demographics may influence the generalisability and applicability of the findings across countries.

Therefore, this study aims to evaluate the psychometric properties of the INAS among PwT2D in the UK by assessing its structural validity, construct validity, internal reliability and test–retest reliability, as well as its predictive validity in 3–6 months.

## 2 METHODOLOGY

### 2.1 Participants

A purposive sample of 260 PwT2D from a diabetes intermediate care team in London participated in this study. The intermediate care team sits between the General Practitioners (GP) and hospital diabetes team, manages PwT2D referred by their GP and provides services such as specialist clinics, telephone and email support for people attending their service, and primary and community care teams. The team updates the GP on any changes in the diabetes care plan after each appointment and discharges people back to their GP for their diabetes management when they meet certain criteria, for example, achieving their target HbA1c.

Participants were PwT2D aged 21 and above who were prescribed medications for diabetes, able to understand English and the study, followed up with the diabetes team and provided informed consent. A subsample of participants who agreed to study follow‐up was recontacted in 2–4 weeks after the baseline questionnaire for test–retest reliability assessment. Those who agreed to study follow‐up, continued receiving care from the diabetes team, had accompanying HbA1c and were not hospitalised since baseline were recontacted in 3–6 months for predictive validity assessment. Participants who were hospitalised were not followed up as acute illnesses may confound their follow‐up HbA1c.

Pregnant women were excluded because their physiological changes could affect diabetes management.

Patient and Public Involvement (PPI) informed the study protocol and participant‐facing documents. This study was approved by the London‐Chelsea Research Ethics Committee (reference number 23/PR/0660).

### 2.2 Measures

The INAS asks PwT2D to rate their agreement with 22 reasons for sometimes stopping diabetes medications in the past 3 months, using a 5‐point Likert scale. The INAS minimises potential social desirability bias by presenting the introduction and reasons neutrally and non‐judgmentally. The initial INAS comprised 2 subscales: ‘*Resisting illness*’, which focuses on medication refusal as unwanted reminders of illness and a desire for normalcy, and ‘*Testing treatment*’, which relates to attempts to manage with less or no medication.^9^ Subsequent validation studies introduced more subscales, including ‘*Mistrust treatment*’,^11^ ‘*Resisting treatment*’,^11^ ‘*Drug‐specific concerns*’,^10^ ‘*General sensitivity to medication*’,^10^ ‘*Sensitivity to medication*’^12^ and ‘*Inconvenience*’.^12^

Demographic information was collected from PwT2D's self‐report on a 5‐item questionnaire, *About you*.

PwT2D's adherence level in the past 3 months was assessed using an adapted single‐item *Visual Analogue Scale* (VAS) on a scale of 0%–100%^13^ and the *Medication adherence report scale‐5* (MARS‐5).^14^ MARS‐5 consists of 5 items measuring the frequency of nonadherence behaviours, such as forgetting and altering doses, on a 5‐point scale, with higher scores indicating better adherence.^14^

Their medication beliefs were examined with the *Beliefs about Medicine Questionnaire* (*BMQ*)‐*specific*, which comprises 10 items divided into Necessity and Concern subscales. Items are rated on a 5‐point scale, with higher scores reflecting stronger beliefs in the necessity of medications and increased concerns about taking them^15^ for the respective subscale.

PwT2D's illness perception was evaluated using the *Brief Illness Perception Questionnaire* (BIPQ), which includes 9 items evaluating aspects such as its consequences, timeline, personal control, treatment control, identity, concern, understanding and emotional response on a 0 to 10 scale.^16^

Their mood was measured with the 2‐item *Patient Health Questionnaire‐2* (PHQ‐2), in which they rated their frequency of depressive symptoms on a 4‐point scale (0 = not at all to 3 = nearly everyday).^17^ Higher scores suggest a greater risk of depression.^17^

All PwT2D completed the INAS and comparator questionnaires at baseline. Some repeated the INAS in 2–4 weeks for test–retest reliability, while some completed the INAS and MARS‐5 in 3–6 months for predictive validity.

Questionnaires were administered in‐person at baseline. Follow‐ups were conducted in‐person during routine appointments or by phone.

Clinical and demographic data, including comorbidities, HbA1c at baseline and follow‐up, and medications, were extracted from PwT2D's electronic medical records.

### 2.3 Statistical analysis

All analyses were performed using Stata18.

A sample size of 260 was determined based on a 95% confidence interval proportion, 40% poor diabetes control,^18^ ±8% precision and a 40% expected loss to follow‐up.^19^

Descriptive statistics were calculated for demographic and clinical characteristics using independent *t*‐test and Wilcoxon Mann‐Whitney test.

Suitability for factor analysis was assessed using a correlation matrix, the Bartlett test of sphericity and Kaiser‐Meyer‐Olkin (KMO) score. Confirmatory factor analysis (CFA) and exploratory factor analysis (EFA) with oblique rotation were performed to evaluate structural validity. The number of retained factors was guided by parallel analysis,^20^ minimum average partial correlation method (MAP)^20^ and Kaiser eigenvalue criterion.^20^ The factor model was iteratively refined by adjusting the number of retained factors,^21^ testing different factor loading thresholds above 0.35,^21^ and removing items with problematic or potential cross‐loadings (based on cross‐loading ratios) or communalities below 0.5^21^ to achieve a theoretically interpretable model. The factors were labelled according to item content.

Internal reliability was evaluated using Cronbach's alpha. Test–retest reliability was assessed using the intraclass correlation coefficient (ICC) with a two‐way random effects model. The 5‐point scale was condensed into a 3‐point scale by combining ‘strongly agree’ with ‘agree’, ‘strongly disagree’ with ‘disagree’, while keeping the ‘neutral’ option unchanged. This modification was made because 29 out of 30 follow‐up questionnaires were administered by phone, making it difficult for participants to distinguish between extreme and moderate responses without having the questionnaires in front of them.

Construct validity was assessed by testing a priori theoretical hypotheses between the INAS factors and comparator measures using Spearman correlations. The INAS factors were expected to negatively correlate with VAS, MARS‐5, BMQ‐necessity, while positively correlating with BMQ‐concern, PHQ‐2 and HbA1c. Illness and treatment perceptions in the BIPQ related to the INAS factors found were hypothesised to demonstrate convergent validity.

Predictive validity, defined as the association of baseline INAS factors with MARS‐5 and HbA1c in 3–6 months, was investigated using quantile and linear regression. Potential covariates for the regressions were identified through Spearman correlations with MARS‐5 and Pearson correlations with HbA1c. Principal component analysis (PCA) was conducted to address potential multicollinearity among the INAS factors. Sensitivity analysis was performed to determine whether participants who were followed up and those who were not followed up in 3–6 months were equivalent at baseline.

## 3 RESULTS

### 3.1 Participants

Participants' characteristics are detailed in Table 1. Their mean age was 60.9 ± 11.7 years. The majority were on both oral and injectable medications (67.69%). Their mean HbA1c was 78 ± 20 mmol/mol (9.3 ± 1.9%). The mean and standard deviation of their INAS responses are shown in Supplementary Material S1.

**TABLE 1:** Participant characteristics.

Variable	*N* (%) or mean ± SD
Age	60.9 ± 11.7
Sex	
Men	128 (49.23)
Women	132 (50.77)
Ethnicity	
White	74 (28.46)
Black/Black British	118 (45.38)
Asian/Asian British	23 (8.85)
Mixed	14 (5.38)
Others	28 (10.77)
Declined to answer	3 (1.15)
Highest education	
No formal education	4 (1.54)
Primary school/lower	18 (6.92)
Secondary school	93 (35.77)
A‐level/diploma	59 (22.69)
Degree/higher	85 (32.69)
Declined to answer	1 (0.38)
Relationship status	
Single	90 (34.62)
Married	112 (43.08)
Separated/divorced/widowed	48 (18.46)
Others	9 (3.46)
Declined to answer	1 (0.38)
Manage own medication	
No	6 (2.31)
Yes	254 (97.69)
Years of diabetes (based on medical record)	
No records	11 (4.23)
≤1 year	12 (4.62)
>1 year, ≤5 years	23 (8.85)
>5 years, <10 years	34 (13.08)
10–19 years	108 (41.54)
≥20 years	72 (27.69)
Medication type	
Oral only	73 (28.08)
Injectables only	11 (4.23)
Oral + injectables	176 (67.69)
Number of chronic diseases	2.39 ± 1.20
Common types of chronic diseases	
Hypertension	133 (51.15)
Chronic kidney disease	57 (21.92)
Ischemic heart disease	32 (12.31)
Depression	31 (11.92)
Osteoarthritis	25 (9.62)
Asthma	20 (7.69)
Baseline HbA1c (mmol/mol)	78 ± 20
Baseline HbA1c (%)	9.3 ± 1.9

Abbreviation: HbA1c, glycated haemoglobin.

Most participants completed the questionnaires with the researcher (*n* = 204, 78.46%), while the remainder self‐administered them at baseline (*n* = 56, 21.54%). Supplementary Material S2 compares self‐administered and interviewer‐administered questionnaire responses and participant characteristics.

The response rate was 82.54%, as 315 PwT2D were approached and 260 agreed to participate.

### 3.2 Structural validity

Suitability for factor analysis was confirmed by significant inter‐item correlations (>0.30), Bartlett test of sphericity (*p* < 0.001) and KMO (0.929).

CFA using previous factor structures^9^, ^10^, ^11^, ^12^ showed a poor fit, with comparative fit indexes (CFI) ranging from 0.794 to 0.854, root mean square error of approximation (RMSEA) from 0.169 to 0.181 and Tucker–Lewis Indexes (TLI) from 0.766 to 0.822.

For EFA, different factor retention methods suggested a varying number of factors: parallel analysis (1), MAP (4) and Kaiser eigenvalue criterion (3). Two items were eliminated for factor loadings <0.40, and two were removed for problematic and potential cross‐loadings. The final 18‐item factor model met factorability criteria: inter‐item correlations >0.30, significant Bartlett test of sphericity (*p* < 0.001) and KMO of 0.929. All retained items showed communalities >0.50, factor loadings >0.40, and had no cross‐loadings.

The four‐factor model in Table 2 accounted for 79.95% of the total variance (Factor 1, 66.49%; Factor 2, 6.22%; Factor 3, 4.81%; and Factor 4, 2.43%). Factors 2–4 were retained despite explaining less variance, given their items' strong loadings (mostly >0.50) and potential to capture additional dimensions of the construct. The four factors were labelled as follows: Factor 1 ‘Resisting illness’ (seven items), Factor 2 ‘Resisting medication’ (five items), Factor 3 ‘Testing treatment’ (three items) and Factor 4 ‘Sensitivity to medication’ (three items).

**TABLE 2:** INAS factor model.

INAS item	Factor loading	Uniqueness
Factor 1 Resisting illness; Cronbach's alpha = 0.96; ICC = 0.10		
Because I want to think of myself as a healthy person again	0.88	0.18
Because I want to lead a normal life again	0.86	0.18
Because it is good not to have to remember	0.81	0.20
Because it is inconvenient to take all the time	0.80	0.27
Because diabetes medication reminds me that I have diabetes	0.74	0.18
Because I worry about becoming dependent on my diabetes medication	0.72	0.24
Because the diabetes medication schedule doesn't fit with my lifestyle	0.68	0.32
Factor 2 Resisting medication; Cronbach's alpha = 0.95; ICC = 0.92		
Because I don't like chemicals in my body	0.79	0.10
Because I think I am on too high a dose	0.59	0.22
Because I don't like the diabetes medication to accumulate in my body	0.60	0.18
Because diabetes medication may affect the body's own natural healing processes	0.63	0.13
Because I think the diabetes medication might become less effective over time	0.48	0.33
Factor 3 Testing treatment; Cronbach's alpha = 0.92; ICC < 0.001		
To see if I really need diabetes medication	0.96	0.02
To see if my diabetes is still there	0.76	0.27
To see if I can do without diabetes medication	0.58	0.24
Factor 4 Sensitivity to medication; Cronbach's alpha = 0.92; ICC = 0.66		
Because my body is sensitive to the effects of diabetes medication	0.94	0.13
Because I don't like the side effects	0.79	0.16
Because the diabetes medication is harsh on my body	0.75	0.29

Abbreviation: ICC, intraclass correlation coefficient.

### 3.3 Internal reliability

The Cronbach's alpha for INAS Factors 1–4 was 0.96, 0.95, 0.92 and 0.92, respectively (Table 2).

### 3.4 Test–retest reliability

Among the 30 participants who repeated the INAS after 2–4 weeks, the ICCs for INAS factors were as follows: ‘Resisting illness’ 0.10, ‘Resisting medication’ 0.92, ‘Testing treatment’ <0.001 and ‘Sensitivity to medication’ 0.66. All participants completed the INAS in‐person at baseline; however, after 2–4 weeks, only one did so; the other 29 completed it over the phone.

### 3.5 Construct validity

Correlations between the INAS and comparator measures are summarised in Table 3. As hypothesised, the INAS exhibited significant negative correlations with VAS, MARS‐5 and positive correlations with BMQ‐concern, supporting convergent validity. ‘Resisting medication’ and ‘Testing treatment’ negatively correlated with BMQ‐necessity, while ‘Resisting illness’ and ‘Sensitivity to medication’ positively correlated with HbA1c. All INAS factors correlated significantly with BIPQ1‐Consequence, BIPQ4‐Treatment control, BIPQ5‐Identity and BIPQ8‐Emotional response. Discriminant validity was demonstrated by the absence of correlations with BIPQ3‐Personal control and BIPQ7‐Understanding. No correlation was observed with PHQ‐2.

**TABLE 3:** Construct validity based on Spearman correlation between INAS factors and other comparator measures.

Construct validity	Factor 1 Resisting illness	Factor 2 Resisting medication	Factor 3 Testing treatment	Factor 4 Sensitivity to medication
VAS	−0.28^a^	‐0.20^b^	‐0.16^b^	‐0.20^b^
MARS‐5	‐0.28^a^	‐0.21^a^	‐0.14^b^	‐0.20^b^
BMQ‐necessity	−0.11	−0.14^b^	−0.16^b^	−0.07
BMQ‐concern	0.31^a^	0.26^a^	0.25^a^	0.20^b^
BIPQ1‐Consequence	0.14^b^	0.16^b^	0.14^b^	0.17^b^
BIPQ2‐Timeline	−0.15^b^	−0.14^b^	−0.09	−0.09
BIPQ3‐Personal control	−0.02	<−0.01	0.02	−0.01
BIPQ4‐Treatment control	−0.14^b^	−0.15^b^	−0.15^b^	−0.14^b^
BIPQ5‐Identity	0.14^b^	0.16^b^	0.16^b^	0.18^b^
BIPQ6‐Concern	0.12	0.14^b^	0.15^b^	0.15^b^
BIPQ7‐Understanding	−0.02	0.03	0.05	0.03
BIPQ8‐Emotional response	0.18^b^	0.15^b^	0.16^b^	0.19^b^
PHQ‐2	0.06	0.03	−0.01	0.06
Hba1c	0.13^b^	0.10	0.08	0.12^b^

Abbreviations: BIPQ, Brief Illness Perception Questionnaire; BMQ, Beliefs about Medicine Questionnaire; HbA1c, glycated haemoglobin; MARS‐5, medication adherence report scale‐5; PHQ‐2, Patient Health Questionnaire‐2; VAS, Visual Analogue Scale.

^a^ *p* < 0.001.

^b^ *p* ≤ 0.05.

### 3.6 Predictive validity

Predictive validity was evaluated in 124 participants who were followed up again in 3–6 months. Some participants could not be followed up because they were discharged by the diabetes team after baseline (*n* = 43), did not attend their follow‐up appointment (*n* = 29) or had their appointment rescheduled more than 6 months later (*n* = 24).

Sensitivity analysis revealed no statistically significant differences at baseline between participants who were followed up and the 136 who were not (Supplementary Material S3).

Quantile regression for follow‐up MARS‐5 was adjusted for baseline MARS‐5, other factors potentially affecting follow‐up HbA1c that showed significant correlation with follow‐up MARS‐5 (Supplementary Material S4), along with medication type and medication changes prior to follow‐up that may influence the association. Linear regression for follow‐up HbA1c was adjusted for baseline HbA1c and medication type that showed significant correlation with follow‐up HbA1c (Supplementary Material S4), along with other relevant covariates, including other factors potentially affecting follow‐up HbA1c, follow‐up MARS‐5 and medication changes prior to follow‐up. The questionnaire administration mode (in‐person vs. phone, self‐ vs. interviewer‐administered) and the time interval between baseline and follow‐up were adjusted for in both regressions. Factor 3 ‘Testing treatment’ showed a trend towards statistical significance with follow‐up MARS‐5 (coefficient = −0.34, *p* = 0.09; in Table 4).

**TABLE 4:** Predictive validity of the INAS (after PCA) on MARS‐5 and HbA1c in 3–6 months.

	MARS‐5^a^		HbA1c^b^	
	Regression coefficient	*p*‐value	Regression coefficient	*p*‐value
Factor 1 Resisting illness	−0.09	0.65	−1.97	0.16
Factor 2 Resisting medication	0.31	0.11	0.65	0.63
Factor 3 Testing treatment	−0.34	0.09	−1.62	0.24
Factor 4 Sensitivity to medication	0.18	0.41	0.28	0.85

Abbreviations: HbA1c, glycated haemoglobin; MARS‐5, medication adherence report scale‐5; PCA, principal component analysis.

^a^ Quantile regression for follow‐up MARS‐5 was adjusted for baseline MARS‐5, other factors potentially affecting follow‐up HbA1c that showed significant correlation with follow‐up MARS‐5, along with medication type and medication changes prior to follow‐up that may influence the association.

^b^ Linear regression for follow‐up HbA1c was adjusted for baseline HbA1c and medication type that showed significant correlation with follow‐up HbA1c, along with other relevant covariates, including other factors potentially affecting follow‐up HbA1c, follow‐up MARS‐5 and medication changes prior to follow‐up. ^a,b^The questionnaire administration and time interval between baseline and follow‐up were adjusted for in both regressions.

## 4 DISCUSSION

This study evaluated the psychometric properties of the INAS, a new measure which quantitatively assesses dimensions of intentional nonadherence identified in previous qualitative adherence research: Resisting illness,^22^ Resisting medications^22^ and Testing treatment.^22^ It also emphasised that ‘Sensitivity to medication’ may be particularly relevant to PwT2D, as this factor also emerged in the validation study conducted among PwT2D in Singapore.^12^

The INAS demonstrated good internal reliability within the recommended range of 0.70 and 0.95.^23^ The Cronbach's alpha coefficients were comparable to the original INAS (0.93–0.95) and higher than earlier factor models (0.84–0.94).^10^, ^11^, ^12^ Higher internal reliability indicates that items within a factor measure the same construct consistently.^23^ An alpha >0.95 may suggest item redundancy.^23^ However, this needs to be balanced with the recommended minimum of three items per factor.^24^ For ‘Resisting illness’ and ‘Resisting medication’, which both had more than three items and an alpha of ≥0.95, item response theory may be employed for potential reduction of the questionnaire length in the future.

Test–retest reliability analysis reported mixed results. The lower ICC for ‘Resisting illness’ and ‘Testing treatment’ likely resulted from different questionnaire administration modes at baseline compared with the follow‐up. Most PwT2D did not have routine clinic appointments within 2–4 weeks, making it difficult to repeat the INAS in‐person. An earlier study found higher ICCs for participants who completed the INAS in‐person at both timepoints (0.69–0.90) than for those with mixed administration modes (0.50–0.66).^12^ Phone administration may yield different results due to the lack of visual cues to aid understanding of the questions, the likelihood of multitasking while answering, satisficing to reduce cognitive effort and potential time constraints.^25^ The lower ICC may not necessarily indicate inadequate reliability of the INAS but reflect the impact of varying administration modes between timepoints.

The INAS factors ‘Resisting illness’ and ‘Resisting medication’ align with qualitative syntheses that highlighted self‐identity and illness perception as key influences on medication adherence in PwT2D.^22^, ^26^ These factors illuminate the complex psychological processes underlying intentional nonadherence, as some PwT2D struggle to accept their diagnosis,^26^ perceive medications as changing their identity,^26^ and associate medication use with being sick.^22^ This resistance may not be limited to people with newly diagnosed type 2 diabetes navigating health changes, but also among those with long‐term diabetes, as over 60% of the participants recruited had diabetes for at least 10 years. The positive correlations between these factors with BMQ‐concern and BIPQ1‐Consequence suggest that this resistance may relate to ongoing concerns about medication side effects and the perceived impact of diabetes. The INAS could help clinicians to identify and quantify these resistance factors, appreciate potential relationships between the INAS factors, illness perceptions and medication beliefs, potentially facilitating their communication with PwT2D and informing targeted interventions to improve adherence.

The INAS factors ‘Testing treatment’ captures PwT2D's tendency to self‐adjust their medication. This behaviour may stem from the preventive nature of diabetes medications, the asymptomatic and slow progression of diabetes. Our study supports this, as ‘Testing treatment’ negatively correlates with BMQ‐necessity and BIPQ4‐Treatment control, while positively correlating with BIPQ5‐Identity. These correlations imply that PwT2D are more likely to test whether they can manage with no or reduced medication if they perceive their treatment as unnecessary, ineffective or if their diabetes symptoms persist. This is consistent with qualitative findings that ongoing symptoms despite adherence can hinder adherence, and that PwT2D self‐adjust medications based on personal criteria, such as missed doses and perceived normal glycaemic level.^26^ The presence of this INAS factor in our validation study, combined with its correlations and qualitative insights,^26^ may reflect PwT2D's desire for autonomy. Quantifying ‘Testing treatment’ through the INAS may enable clinicians to tailor their approach by balancing medical guidance with PwT2D's autonomy and providing early intervention, considering its borderline significant association with adherence in 3–6 months.

The INAS factor ‘Sensitivity to medication’ may illustrate the interaction between physiological and psychological influences in PwT2D. Physiologically, chronic kidney disease affects 30% of the PwT2D in England,^27^ impairing renal clearance of many diabetes medications, such as insulin, metformin and sulfonylureas, which increase the risk of side effects.^28^ Psychologically, although no correlation was found with PHQ‐2, a significant positive correlation with BIPQ8‐Emotional response suggests that PwT2D who are more emotionally affected by diabetes may perceive greater sensitivity to medication. This may be partly explained by the symptom perception model, which posits that negative emotions can heighten awareness of and sensitivity to physical sensations,^29^ including medication effects. The presence of this INAS factor in our study and the previous Singapore^12^ study underscores its importance among PwT2D and highlights its potential as a target for future adherence interventions.

Our longitudinal study design and the use of biomarker and self‐report measures were strengths that allowed us to explore different dimensions of nonadherence and the temporal effects of the INAS on adherence and HbA1c, offering insights for potential early targeted intervention. The high initial participation rate was likely contributed to by the diabetes team's support, which helped identify potential participants in the shared waiting room with the general practices.

This study was constrained by practical and resource limitations. Study follow‐up was hampered by inconsistent questionnaire administration modes. While questionnaires were administered in‐person at baseline, follow‐up questionnaire administration was less consistent, as participants may not have had scheduled clinic appointments at the study time point and could be missed or uncontactable due to unexpected changes in their appointments. Nonetheless, the 124 participants in the follow‐up sample remained sizeable and relatively well represented, with no significant baseline differences from the 136 participants who were not followed up. The possible influence of different questionnaire administration modes was also accounted for in our predictive validity analysis.

The non‐significant results in the predictive validity analysis may be contributed by the complex and dynamic nature of factors influencing MARS‐5 and HbA1c over time. Changes in factors, such as diet and physical activity, could not be captured in our study due to response burden from the current questionnaire length but may be considered in future longitudinal studies.

While the INAS has been validated among PwT2D in Singapore, differences in culture and healthcare context may have contributed to the different factor structure observed among PwT2D in the UK. This highlights the importance of validating the questionnaires in different clinical groups and cultural contexts.

The varying INAS factor structures across different clinical and demographic populations suggest that a review may be beneficial to establish a more universally applicable structure for facilitating comparisons.

## 5 CONCLUSION

The INAS performed well on a number of psychometric indicators and may serve as a helpful tool for clinicians in identifying specific drivers of intentional nonadherence among PwT2D. This could support the development of tailored interventions to target specific barriers.

## AUTHOR CONTRIBUTIONS

**Vivien Teo:** Conceptualization, data curation, formal analysis, investigation, methodology, project administration, writing—original draft, review and editing. **Anna Hodgkinson, John Weinman, Mark Chamley and Kai Zhen Yap:** Conceptualization, methodology, supervision, writing—review and editing.

## FUNDING INFORMATION

This study did not receive any specific grant from funding agencies in the public, commercial or not‐for‐profit sectors. The declaration of this original work is approved by all authors.

## CONFLICT OF INTEREST STATEMENT

All authors declare that they have no conflict of interest.

## Supporting information


Data S1.

## Data Availability

The data that supports the findings of this study are available in the Supplementary Material of this article.
