# Supplementary material for: A validation study of the Intentional Nonadherence Scale among people with type 2 diabetes in the United Kingdom
Source: Diabet Med. 2025 Apr 5;42(6):e70040. doi: 10.1111/dme.70040 (PMC12080987; doi:10.1111/dme.70040)
Supplement: Supplementary file 1 — Data S1. [file DME-42-e70040-s001.docx]

# Supplementary material 1: Mean and standard deviation for the INAS items

| I sometimes stop taking my diabetes medication in the past 3 months | Mean ± Standard deviation |
| --- | --- |
| 1. To see if my diabetes is still there | 1.77 ± 0.62 |
| 2. To see if I can do without diabetes medication | 1.87 ± 0.75 |
| 3. To see if I really need diabetes medication | 1.86 ± 0.75 |
| 4. Because I am not convinced that the diabetes medication is really right for me* | 1.89 ± 0.78 |
| 5. Because I am not sure that the doctor chose the right diabetes medication for me* | 1.84 ± 0.69 |
| 6. To give my body a rest from the diabetes medication* | 1.86 ± 0.71 |
| 7. Because the diabetes medication is harsh on my body | 1.95 ± 0.80 |
| 8. Because I don’t like the diabetes medication to accumulate in my body | 1.83 ± 0.68 |
| 9. Because my body is sensitive to the effects of diabetes medication | 1.93 ± 0.80 |
| 10. Because I don’t like the side effects | 1.98 ± 0.86 |
| 11. Because I don’t like chemicals in my body | 1.89 ± 0.76 |
| 12. Because diabetes medication may affect the body’s own natural healing processes | 1.90 ± 0.76 |
| 13. Because I think I am on too high a dose | 1.87 ± 0.71 |
| 14. Because I think the diabetes medication might become less effective over time | 1.90 ± 0.71 |
| 15. Because I worry about becoming dependent on my diabetes medication | 1.90 ± 0.74 |
| 16. Because I want to think of myself as a healthy person again | 1.88 ± 0.71 |
| 17. Because diabetes medication reminds me that I have diabetes | 1.84 ± 0.67 |
| 18. Because I want to lead a normal life again | 1.92 ± 0.77 |
| 19. Because it is good not to have to remember | 1.85 ± 0.71 |
| 20. Because it is inconvenient to take all the time | 1.93 ± 0.76 |
| 21. Because the diabetes medication schedule doesn’t fit with my lifestyle | 1.86 ± 0.66 |
| 22. Because I don’t think the diabetes medication is worth it* | 1.78 ± 0.61 |

*Items 4, 5, 6 and 22 were not included in the final INAS factor structure.

# Supplementary material 2: Comparison of self-administered and interviewer-administered questionnaires and participant characteristics

| Variable | Number (%) or Mean ± Standard deviation or Median (Interquartile range) | | |
| --- | --- | --- | --- |
|  | Self  (n=56) | Interviewer  (n =204) | p-value |
| Age | 57.32 ± 11.65 | 61.91 ± 11.51 | **<0.01** |
| Sex (based on the biological attribute) |  | | 0.28 |
| Male | 24 | 104 |  |
| Female | 32 | 100 |  |
| Ethnicity |  |  | **<0.01** |
| White | 28 | 46 |  |
| Black | 20 | 98 |  |
| Asian | 3 | 20 |  |
| Mixed | 2 | 12 |  |
| Others | 2 | 26 |  |
| Declined to report | 1 | 2 |  |
| Highest education |  |  | **0.01** |
| No formal education | 1 | 3 |  |
| Primary school/ lower | 0 | 18 |  |
| O-level/ Secondary school | 15 | 78 |  |
| A-level/diploma | 11 | 48 |  |
| Degree/higher | 29 | 56 |  |
| Declined to report | 0 | 1 |  |
| Relationship status |  |  | 0.71 |
| Single | 22 | 68 |  |
| Married | 21 | 91 |  |
| Separated/ divorced/ widowed | 12 | 36 |  |
| Others | 1 | 8 |  |
| Declined to report | 0 | 1 |  |
| Manage own medication |  |  | 0.19 |
| Yes | 0 | 6 |  |
| No | 56 | 198 |  |
| Years of T2DM (based on medical records) |  |  | 0.25 |
| No records | 3 | 8 |  |
| ≤ 1 year | 3 | 9 |  |
| >1 year, ≤ 5 years | 8 | 15 |  |
| >5 years, <10 years | 9 | 25 |  |
| 10-19 years | 24 | 84 |  |
| ≥ 20 years | 9 | 63 |  |
| Medication type |  |  | **0.04** |
| Oral only | 22 | 51 |  |
| Injectables only | 0 | 11 |  |
| Oral + injectables | 34 | 142 |  |
| Number of chronic diseases (including diabetes) | 2.29 ± 1.00 | 2.42 ± 1.25 | 0.47 |
| Baseline HbA1c (mmol/mol) | 77.63 ± 22.70 | 78.09 ± 19.81 | 0.88 |
| Baseline HbA1c (%) | 9.25 ± 2.08 | 9.30 ± 1.81 | 0.86 |
| Visual analogue scale on extent of adherence | 100 (100-80) | 90 (100-80) | **<0.01** |
| INAS factor 1 | 11.61 ± 6.44 | 13.59 ± 3.71 | **<0.01** |
| INAS factor 2 | 7.93 ± 4.39 | 9.78 ± 2.83 | **<0.001** |
| INAS factor 3 | 4.41 ± 2.32 | 5.79 ± 1.77 | **<0.001** |
| INAS factor 4 | 5.07 ± 2.99 | 6.08 ± 2.00 | **<0.01** |
| Baseline MARS-5 | 23 (25-20.5) | 24 (25-23) | **<0.01** |
| BMQ necessity | 17.75 ± 4.68 | 17.59 ± 3.24 | 0.77 |
| BMQ concern | 12.02 ± 4.34 | 13.97 ± 3.85 | **<0.01** |
| BIPQ1 consequence | 5.52 ± 2.21 | 6.30 ± 2.76 | **0.05** |
| BIPQ2 timeline | 7.59 ± 2.25 | 7.93 ± 2.43 | 0.34 |
| BIPQ3 personal control | 7.30 ± 1.82 | 7.04 ± 2.42 | 0.45 |
| BIPQ4 treatment control | 8.48 ± 1.46 | 8.12 ± 2.07 | 0.23 |
| BIPQ5 Identity | 5.41 ± 2.18 | 6.27 ± 2.78 | **0.03** |
| BIPQ6 Concerns | 7.68 ± 2.25 | 7.83 ± 2.60 | 0.69 |
| BIPQ7 Understanding | 7.79 ± 1.79 | 7.98 ± 2.05 | 0.52 |
| BIPQ8 Emotional response | 4.80 ± 3.19 | 5.65 ± 3.41 | 0.10 |
| PHQ-2 | 1 (3-0) | 0 (2-0) | **0.03** |

For ease of reference, all BIPQ and BINAS were presented in terms of mean +/- SD regardless of normality.

# Supplementary material 3: Sensitivity analysis between participants were and were not followed up in 3-6 months

| Variable | Number (%) or Mean ± Standard deviation or Median (Interquartile range) | | |
| --- | --- | --- | --- |
|  | No follow-up (n=136) | Follow-up (n=124) | p-value |
| Age | 60.7 ± 12.6 | 61.2 ± 10.7 | 0.71 |
| Sex (based on the biological attribute) |  |  | 0.33 |
| Male | 63 (49.2) | 65 (50.8) |  |
| Female | 73 (55.3) | 59 (44.7) |  |
| Ethnicity |  |  | 0.26 |
| White | 36 (48.6) | 38 (51.4) |  |
| Black | 66 (55.9) | 52 (44.1) |  |
| Asian | 9 (39.1) | 14 (60.9) |  |
| Mixed | 9 (64.3) | 5 (35.7) |  |
| Others | 13 (46.4) | 15 (53.6) |  |
| Declined to report | 3 (100.0) | 0 |  |
| Highest education |  |  | 0.69 |
| No formal education | 3 (75.0) | 1 (25.0) |  |
| Primary school/ lower | 10 (55.6) | 8 (44.4) |  |
| O-level/ Secondary school | 46 (49.5) | 47 (50.5) |  |
| A-level/diploma | 34 (57.6) | 25 (42.4) |  |
| Degree/higher | 42 (49.4) | 43 (50.6) |  |
| Declined to report | 1 (100.0) | 0 |  |
| Relationship status |  |  | 0.17 |
| Single | 52 (57.8) | 38 (42.2) |  |
| Married | 51 (45.5) | 61 (54.5) |  |
| Separated/ divorced/ widowed | 25 (52.1) | 23 (47.9) |  |
| Others | 7 (77.8) | 2 (22.2) |  |
| Declined to report | 1 (100.0) | 0 |  |
| Manage own medication |  |  | 0.91 |
| Yes | 3 (50.0) | 3 (50.0) |  |
| No | 133 (52.4) | 121 (47.6) |  |
| Years of diabetes |  |  | 0.08 |
| No records | 8 (72.7) | 3 (27.3) |  |
| ≤ 1 year | 5 (41.7) | 7 (58.3) |  |
| >1 year, ≤ 5 years | 18 (78.3) | 5 (21.7) |  |
| >5 years, <10 years | 18 (52.9) | 16 (47.1) |  |
| 10-19 years | 52 (48.1) | 56 (51.9) |  |
| ≥ 20 years | 35 (48.6) | 37 (51.4) |  |
| Medication type |  |  | 0.10 |
| Oral only | 46 (63.0) | 27 (37.0) |  |
| Injectables only | 5 (45.5) | 6 (54.5) |  |
| Oral + injectables | 85 (48.3) | 91 (51.7) |  |
| Number of chronic diseases (including diabetes) | 2.41 ± 1.26 | 2.36 ± 1.14 | 0.74 |
| Baseline HbA1c (mmol/mol) | 77.45 ± 23.09 | 78.58 ± 17.10 | 0.66 |
| Baseline HbA1c (%) | 9.24 ± 2.11 | 9.35 ± 1.57 | 0.66 |
| Visual analogue scale on extent of adherence | 100 (100-80) | 100 (100-80) | 0.91 |
| INAS factor 1 | 13.10 ± 4.28 | 13.24 ± 4.75 | 0.79 |
| INAS factor 2 | 9.29 ± 3.20 | 9.48 ± 3.43 | 0.65 |
| INAS factor 3 | 5.48 ± 2.08 | 5.51 ± 1.87 | 0.90 |
| INAS factor 4 | 5.96 ± 2.43 | 5.76 ± 2.11 | 0.47 |
| Baseline MARS-5 | 24 (25-22) | 24 (25-22) | 0.38 |
| BMQ necessity | 17.61 ± 3.65 | 17.64 ± 3.54 | 0.95 |
| BMQ concern | 13.40 ± 4.25 | 13.71 ± 3.80 | 0.54 |
| BIPQ1 consequence | 5.91 ± 2.82 | 6.37 ± 2.47 | 0.17 |
| BIPQ2 timeline | 7.88 ± 2.41 | 7.83 ± 2.39 | 0.86 |
| BIPQ3 personal control | 7.19 ± 2.19 | 6.99 ± 2.43 | 0.49 |
| BIPQ4 treatment control | 8.08 ± 2.03 | 8.33 ± 1.88 | 0.31 |
| BIPQ5 Identity | 6.10 ± 2.81 | 6.06 ± 2.55 | 0.91 |
| BIPQ6 Concerns | 7.73 ± 2.68 | 7.88 ± 2.35 | 0.63 |
| BIPQ7 Understanding | 8.08 ± 1.88 | 7.78 ± 2.10 | 0.23 |
| BIPQ8 Emotional response | 5.38 ± 3.41 | 5.56 ± 3.35 | 0.65 |
| PHQ-2 | 1 (2-0) | 0 (2-0) | 0.65 |

For ease of reference, all BIPQ and BINAS were presented in terms of mean +/- SD regardless of normality.

# Supplementary material 4: Correlation between potential covariates and MARS-5 and HbA1c in 3-6 months

| Potential covariates | Follow-up MARS-5  (Spearman correlation) | | Follow-up HbA1c  (Pearson correlation) | |
| --- | --- | --- | --- | --- |
|  | Correlation coefficient | p-value | Correlation coefficient | p-value |
| Age | 0.12 | 0.19 | -0.05 | 0.60 |
| Gender | 0.05 | 0.60 | 0.15 | 0.10 |
| Ethnicity | 0.02 | 0.83 | 0.12 | 0.20 |
| Highest education level | -0.14 | 0.13 | -0.13 | 0.15 |
| Relationship status | -0.10 | 0.28 | -0.08 | 0.40 |
| Allergy status/ adverse drug reaction | 0.03 | 0.78 | -0.07 | 0.46 |
| Managing own medication | 0.11 | 0.24 | 0.13 | 0.16 |
| Years of diabetes | -0.06 | 0.50 | 0.08 | 0.37 |
| Number of chronic diseases | -0.02 | 0.84 | 0.08 | 0.38 |
| Medication type | -0.02 | 0.85 | **0.18** | **0.04** |
| Medication changes prior to follow-up | 0.14 | 0.13 | 0.02 | 0.81 |
| Other factors potentially affecting follow-up HbA1c | **-0.20** | **0.03** | -0.03 | 0.75 |
| Baseline MARS-5 | **0.46** | **0.00** | -0.07 | 0.46 |
| Baseline HbA1c | -0.06 | 0.53 | **0.52** | **0.00** |
| Follow-up MARS-5 | - | - | -0.08 | 0.38 |

HbA1c: Glycated haemoglobin; MARS-5: Medication adherence report scale-5
